# “IT TAKES A SPECIAL PERSON”: EXPLORING NURSING STUDENTS’ END-OF-LIFE CARE EXPERIENCES AND ITS INFLUENCE ON EMOTIONS

**DOI:** 10.1093/geroni/igae098.4325

**Published:** 2024-12-31

**Authors:** Kalei Kowalchik

**Affiliations:** The Pennsylvania State University, Olyphant, Pennsylvania, United States

## Abstract

Navigating end-of-life care as a nursing student can results in intense emotions such as grief and despair and there is limited research on undergraduate nursing students’ thoughts and experiences on the need for emotional support resources when caring for patients and families at the end of life. To explore to explore and understand what end-of- life care experiences influence undergraduate nursing students’ emotions. A qualitative descriptive design study was used to interview third- and fourth-year nursing students. Purposive sampling techniques were used to recruit participants. Demographic data was collected via Qualtrics, and interviews were held via face-to-face (n=4) and Zoom (n=17). Data were transcribed verbatim, de-identified, and analyzed for themes. Results show that providing care to dying patients and their families is emotionally impactful to students and creates self-awareness about death and mortality. Students’ personal, educational, occupational, and clinical experiences caring for dying patients influences how they process and regulate their emotions when professionally caring for dying patients and their families. Additionally, these experiences are influential to how they interact with death and dying topics in their personal lives. Caring for dying patients and exposure to death as a nursing student is emotionally impactful to students as an emerging professional and as a person. Nurse educators should integrate supportive resources for students caring for dying patients to help develop students’ resilience, emotional intelligence, and self-awareness when providing EOL care.

